# Intraoral ultrasonography in the assessment of DOI in oral cavity squamous cell carcinoma: a comparison with magnetic resonance and histopathology

**DOI:** 10.1007/s00405-020-06421-w

**Published:** 2020-10-21

**Authors:** Marta Filauro, Francesco Missale, Filippo Marchi, Andrea Iandelli, Andrea Luigi Camillo Carobbio, Francesco Mazzola, Giampiero Parrinello, Emanuele Barabino, Giuseppe Cittadini, Davide Farina, Cesare Piazza, Giorgio Peretti

**Affiliations:** 1 IRCCS Policlinico San Martino, Largo Rosanna Benzi, 10, 16132 Genoa, Italy; 2 Interdisciplinary Department of Surgical and Integrated Diagnostic Sciences (DISC), University of Genoa, Genoa, Italy; 3 Department of Experimental Medicine (DIMES), University of Genoa, Genoa, Italy; 4 Department of Molecular and Translational Medicine, University of Brescia, Brescia, Italy; 5 Department of Diagnostic Radiology, IRCCS Policlinico San Martino, Genoa, Italy; 6 Department of Radiology, University of Brescia, Brescia, Italy; 7 Department of Otorhinolaryngology, Maxillofacial, and Thyroid Surgery, Fondazione IRCCS, National Cancer Institute of Milan, University of Milan, Milan, Italy

**Keywords:** Mouth, Neoplasm, Magnetic resonance imaging, Ultrasonography, Depth of invasion, Head and neck

## Abstract

**Objective:**

The first-line therapeutic approach for oral cavity squamous cell carcinoma (OCSCC) is complete surgical resection. Preoperative assessment of depth of invasion (cDOI) is crucial to plan the surgery. Magnetic resonance (MR) and intraoral ultrasonography (IOUS) have been shown to be useful tools for assessment of DOI. The present analysis investigates the accuracy of MR and IOUS in evaluating DOI in OCSCC compared to histological evaluation (pDOI).

**Materials and methods:**

Forty-nine previously untreated patients with cT1-T3 OCSCC were reviewed. Nine patients were staged with MR alone, 10 with IOUS alone, and 30 with both MR and IOUS.

**Results:**

Mean difference between cDOI_MR_ and pDOI values of 0.2 mm (95% CI − 1.0–1.3 mm) and between cDOI_IOUS_ and pDOI of 0.3 mm (95% CI − 1.0–1.6 mm). Spearman *R* between cDOI_MR_ and pDOI was *R* = 0.83 and between cDOI_IOUS_ and pDOI was *R* = 0.76. Both radiological techniques showed high performance for the correct identification, with the optimum cut-off of 5 mm, of patients with a pDOI ≥ 4 mm and amenable to a neck dissection, with an AUC of 0.92 and 0.82 for MR and IOUS, respectively.

**Conclusion:**

Both examinations were valid approaches for preoperative determination of DOI in OCSCC, although with different cost-effectiveness profiles and indications.

**Electronic supplementary material:**

The online version of this article (10.1007/s00405-020-06421-w) contains supplementary material, which is available to authorized users.

## Introduction

Head and neck cancer is the 6th most common malignancy worldwide, and oral cavity tumors account for nearly one-third of the tumors [1]. Squamous cell carcinoma (SCC) is the most frequent histotype: it usually arises in the mobile tongue, followed by the lip, floor of the mouth, and buccal mucosa. There is a male preponderance and the vast majority of patients are heavy smokers and alcohol abusers.

Novel relevant changes in the T classification of oral cavity SCC (OCSCC) were brought about by the 8th Edition of the AJCC UICC TNM Staging System [2]. Neoplastic depth of invasion (DOI) was introduced as one of the main aspects to be considered during tumor staging. DOI is defined as “the deepest invasion of tumor in the tissue from the mucosal surface or from a theoretical reconstructed normal mucosal line” [3]. Thus, it profoundly differs from “tumor thickness” (TT), the latter being defined as the distance of the tumor surface from the deepest level of invasion [4]. Consequently, DOI may result in appreciably less TT in exophytic lesions and higher in ulcerated ones, while the two values may correspond in case of fundamentally flat tumors. It is crucial to precisely evaluate the tendency of the tumor to infiltrate healthy tissue since this characteristic reflects the presence of regional lymph node metastases. Thus, recognizing which radiological examination performs better in giving precise preoperative assessment of DOI is of utmost value. However, definitive DOI estimation can be obtained only from measures performed at final histopathological examination (per se subjected to inconsistent variations in terms of tissue shrinkage due to chemical fixation), even if the possibility to have an adequate preoperative evaluation of this parameter allows the surgeon to properly devise the surgical resection (e.g. choosing between transoral resection or compartmental surgery with flap reconstruction) as well as the need for elective neck dissection (strongly suggested for OCSCC with DOI ≥ 4 mm) [5–7]. Moreover, preoperative radiologic measurement of DOI gives the surgeon essential information to tailor the resection within free margins, with particular reference to the deepest one, which represents a significant treatment-related predictor in terms of disease-free survival and loco-regional control [8].

This study aims to identify the best radiological examination for patients with OCSCC in assessing tumor staging, with special reference to DOI, and, consequently, to tailor the best surgical treatment in terms of oncological radicality on T and N sites. In particular, a direct comparison was performed between magnetic resonance (MR), one of the gold standards in modern OCSCC preoperative imaging [9], and intraoral ultrasonography (IOUS), an emerging technique with a new profile of interest.

## Materials and methods

We retrospectively collected data from 49 patients affected by cT1-T3 OCSCC treated at our institution between April 2016 and October 2019.

Inclusion criteria encompassed: (1) clinical evidence and biopsy confirmation of a previously-untreated OCSCC; (2) preoperative evaluation of the lesion by IOUS and/or MR; (3) complete excision of the lesion to obtain its final histopathological evaluation and tumor measurement. Tumor clinical DOI (cDOI) was measured on MR (cDOI_MR_) and IOUS (cDOI_IOUS_) images and compared with the final histopathological measure (pDOI).

Preoperative radiological assessment of tumor size, together with clinical inspection and palpation, was used for T staging and proper discussion at the multidisciplinary tumor (MDT) board.

The superficial width of the lesion was preoperatively evaluated in the outpatient clinic by rigid endoscopy under white light (WL) and narrow-band imaging (NBI, Olympus Medical System Corporation, Tokyo, Japan). The deep extension of the tumor was assessed by imaging (IOUS and/or MR) performed by dedicated radiologists. A supplementary detailed study of the neck was routinely performed with the US, possibly in association with fine-needle aspiration cytology in case of suspected lymph node metastases.

Intraoperative rigid endoscopy with 0**°** telescopes under WL and NBI was repeated with the patient under general anesthesia to establish the appropriate surgical incision for radical excision [10]. All patients underwent complete surgical resection of the primary tumor with or without neck dissection according to NCCN guidelines [11]. For final data analysis, all tumors were homogeneously reclassified according to the 8th Edition of the AJCC UICC TNM Staging System [12]. Patients with biological risk factors such as perineural invasion, angioembolization, multiple lymph nodes metastases, pT4a category, poor differentiation, and/or extracapsular spread underwent adequate adjuvant treatment after MDT board discussion.

The entire cohort of patients received follow-up clinical examination every 2 months during the first 2 years, every 3 months during the third year, every 6 months in the fourth and fifth years, and then annually [11]. Radiological follow-up was performed twice in the first year and then annually for at least three years, even in the absence of suspicious clinical findings.

### MR

**Fig. 1:**
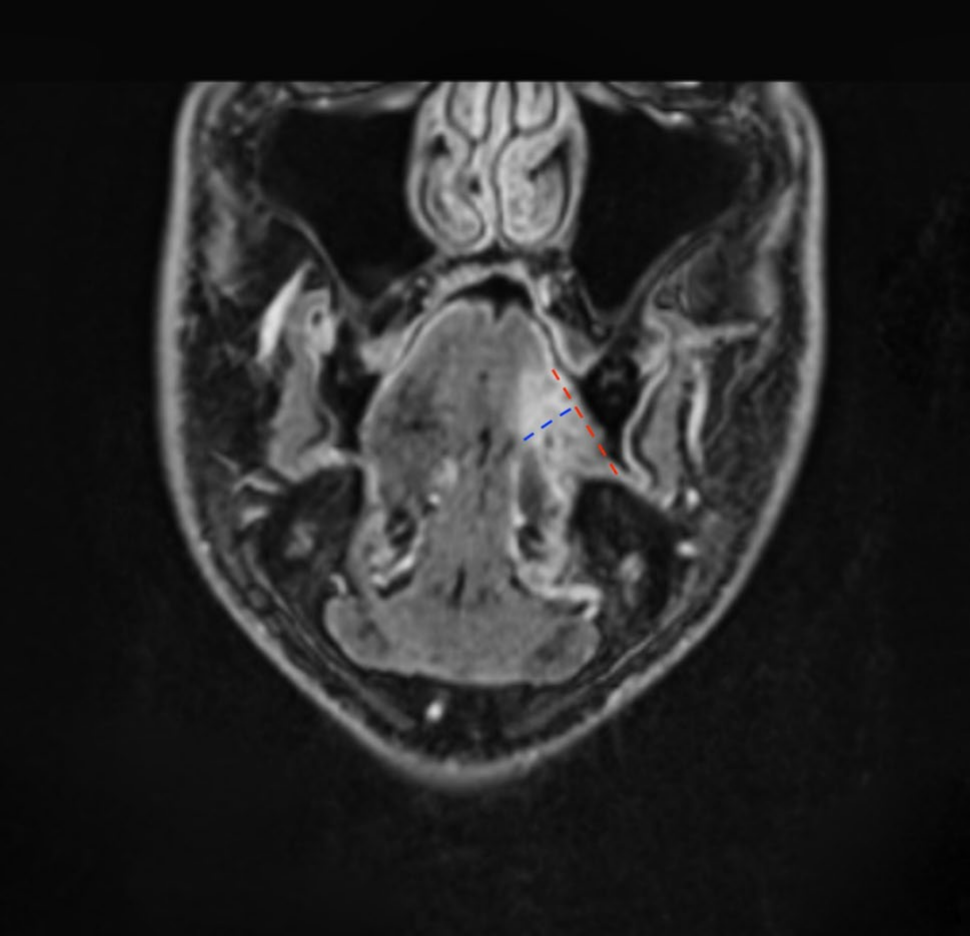
Coronal-reformatted 3D T1-GRE acquisition showing an ulcerated squamous cell carcinoma of the oral tongue. cDOI_MR_ was measured (blue dashed line) perpendicularly to the mucosal plane (red dashed line)

MR was performed with a 1.5-T scanner (Aera, Siemens, Erlangen, Germany) and a 3.0-T scanner (Prisma, Siemens, Erlangen, Germany) with the manufacturer’s phased-array head and neck coils. MR protocol included: axial, sagittal and coronal high-resolution turbo-SE T2 weighted sequences, slice thickness 3 mm, axial turbo-SE T1 weighted sequences, coronal turbo-STIR, and axial echo-planar diffusion-weighted imaging (DWI) sequence with b values of 50 and 800 s/mm^−2^. Axial BLADE T2 weighted images with fat saturation were achieved in case of motion-related artifacts and a 3D fat saturated VIBE sequence with an isotropic pixel of 0.7 mm after paramagnetic contrast agent injection. Apparent diffusion coefficient (ADC) maps were automatically produced for each exam using a mono-exponential model. Two dedicated head and neck radiologists measured DOI independently, choosing the best sequence and plane in which tumor infiltration was depicted (Fig. 1). DOI was measured by drawing a plumb line to that connecting two edges of normal perilesional mucosa up to the most distant front of infiltration of the tumor deep in the tissue. If there was any mismatch between the measures reported by the two radiologists, the highest DOI value was considered and recorded as cDOI_MR_.

### IOUS

**Fig. 2:**
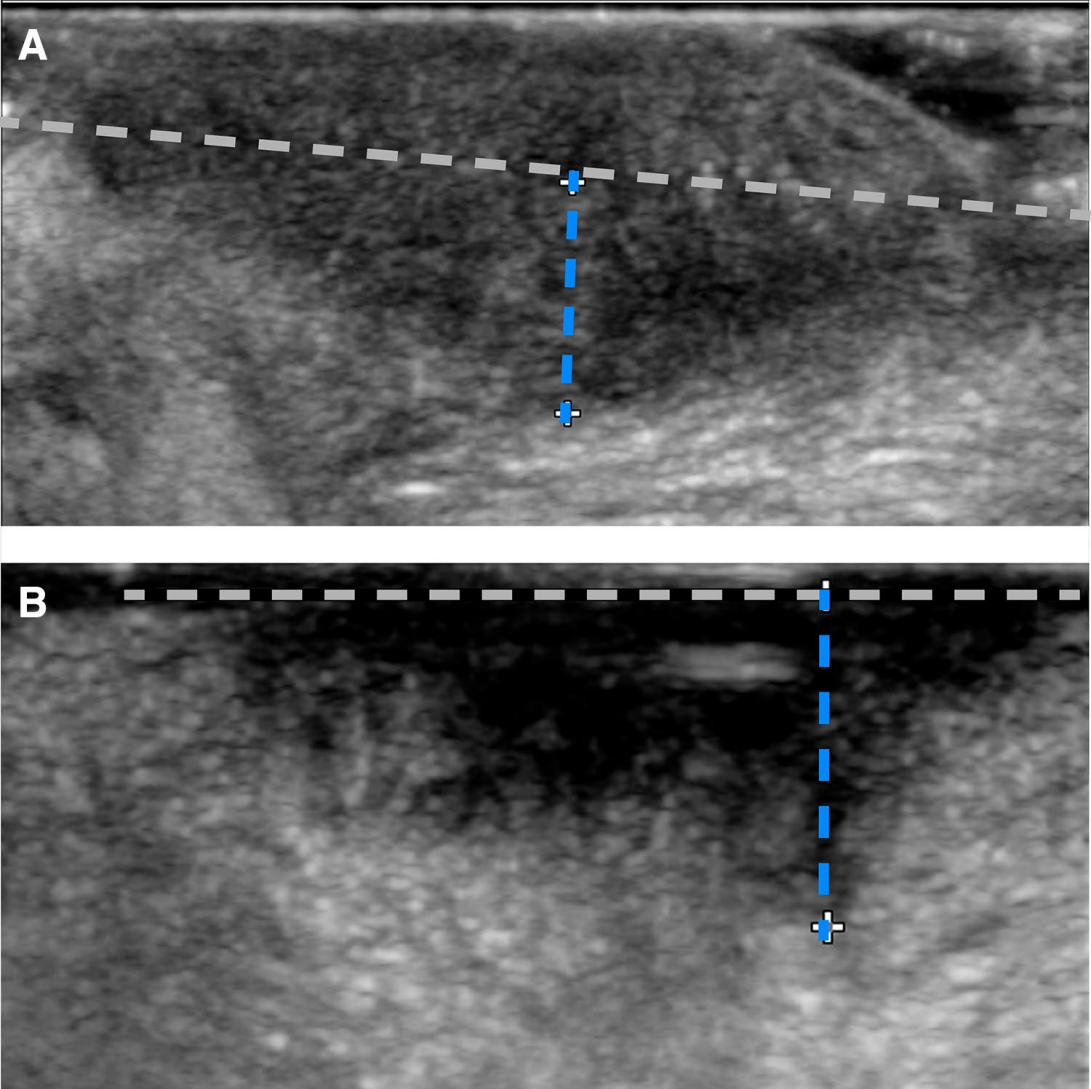
**a** DOI estimation (blue dashed line) in an exophytic SCC of the lateral border of the oral tongue. **b** Estimation of cDOI_IOUS_ in tongue carcinoma (blue dashed line). The dashed grey line represents the mucosal plane. The lesion was ulcerated: in such cases, a small amount of gel may be helpful to prevent air artifacts

IOUS was performed by a dedicated head and neck radiologist using a hockey-stick high frequency (15–7 MHz) probe (Philips Healthcare, Philips North America Corporation, Andover, MA, USA) (Fig. 2a, b). A latex-free cover was filled with gel and placed on the probe. The lesion was fully explored to individuate the point of deepest infiltration. Next, to accurately measure its DOI, the probe was orientated along a plane perpendicular to the mucosal surface using light pressure in order not to alter tumor relationships with the mucosal plane or surrounding tissues. Several US images of the same lesion were recorded to obtain multiple measures of cDOI_IOUS_. The highest measure was considered for comparison with the corresponding cDOI_MR_.

### Statistical analysis

Qualitative variables were described as absolute and relative frequencies; standard descriptive statistics were used for continuous variables, expressing means, medians, ranges and standard deviations. Shapiro–Wilk test was applied, testing normality distribution of continuous variables. Correlation analysis was performed using Spearman’s rank correlation estimating confidence intervals by bootstrap with 1000 resamples, and paired comparison between methods of DOI measurement were tested by Wilcoxon Signed Rank test, as appropriate. The agreement in allocating tumors in different T categories within radiological techniques and compared to the pathological result was investigated by weighted Cohen’s kappa. Cut-off optimization for cDOI_MR_ and cDOI_IOUS_ for the prediction of a target pDOI ≥ 4 mm was performed by Receiver operating characteristic (ROC) curves analysis and maximization of Youden’s index. In all analyses, two-tail tests with a significance level of 5% were applied. R (version 3.6.3, R Foundation for Statistical Computing, Vienna, Austria) with the packages was used for statistical analysis and graphs drawing.

## Results

Forty-nine patients (22 females, 27 males; mean age, 65.6 years; range, 22–95) met inclusion criteria. Nine (18.4%) patients were staged by MR alone, 10 (20.4%) by IOUS alone, and 30 (61.2%) with both MR and IOUS (Table 1). Primary subsite was buccal mucosa in 6 patients, the floor of mouth in 4, and oral tongue in 39 (Table 1). The mean time elapsed between radiological imaging and surgery was 23.3 days (range, 1–67) for MR and 23.1 days (range, 1–118) for IOUS (Table 1). Further clinical and pathological T and N categories are reported in Table 1.

**Table 1:** Summary statistics of demographic and clinical variables

	Overall (*N* = 49)
Age	
Mean (SD)	65.6 (15.8)
Median [Min, Max]	65.0 [22.0, 95.0]
Sex	
F	22 (44.9%)
M	27 (55.1%)
Site	
Buccal mocosa	6 (12.2%)
Tongue	39 (79.6%)
Floor of the mouth	4 (8.2%)
cT category	
T1	16 (32.7%)
T2	20 (40.8%)
T3	13 (26.5%)
cN category	
N0	39 (79.6%)
N1	2 (4.1%)
N2b	5 (10.2%)
N2c	1 (2.0%)
N3b	2 (4.1%)
pT category	
T1	15 (30.6%)
T2	21 (42.9%)
T3	13 (26.5%)
pN category	
N0	18 (36.7%)
N1	5 (10.2%)
N2b	2 (4.1%)
N2c	2 (4.1%)
N3b	4 (8.2%)
Nx	18 (36.7%)
Δ (Date surgery–Date MR)	
Mean (SD)	23.3 (16.2)
Median [Min, Max]	20.0 [1.00, 67.0]
Missing	10 (20.4%)
Δ (Date surgery–Date IOUS)	
Mean (SD)	23.1 (21.8)
Median [Min, Max]	22.0 [1.00, 118]
Missing	9 (18.4%)

**Fig. 3:**
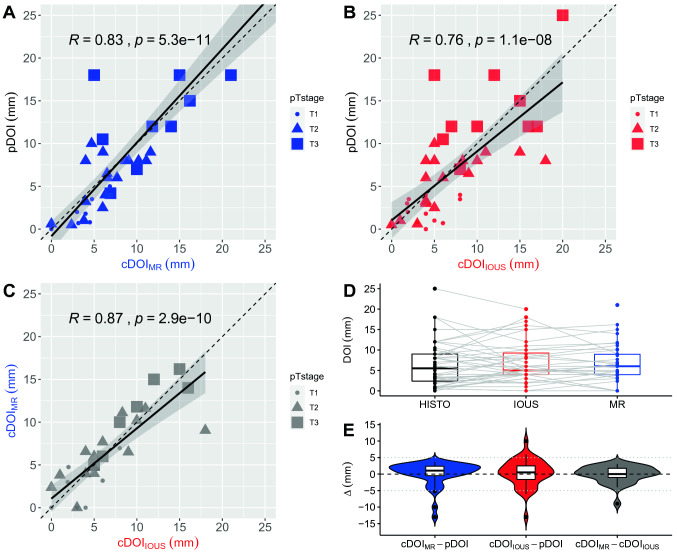
Scatter plots showing the comparisons of pDOI with cDOI_MR_ (**a**) or cDOI_IOUS_ (**b**) and between cDOI_MR_ and cDOI_IOUS_ (**c**); plotted black linear regression line with 95% CI bands in shadow gray; pT categories reported. Box plots showing paired DOI measures (**d**) and violin plots reporting differences distributions comparing the DOI assessed by different techniques

In the entire cohort, the mean value of DOI according to the different examinations was: cDOI_MR_, 7.2 mm (95% confidence interval [95% CI] 5.3–9.2 mm); cDOI_IOUS_, 7.0 mm (95% CI 5.4–8.6 mm); pDOI, 7.3 mm (95% CI 5.2–9.4 mm) (Table 2). Spearman’s rank correlation coefficient between MR and HIST was *R* = 0.83 (95% CI 0.64–0.94, *p* < 0.0001), between IOUS and HIST it was *R* = 0.76 (95% CI 0.59–0.87, *p* < 0.0001) and in-between MR and IOUS *R* = 0.87 (95% CI 0.74–0.94, *p* < 0.0001) indicating good correlations, as reported in Fig. 3a–c and Table 3.

**Table 2:** Summary statistics of DOI measurements and differences among measurement techniques

	Overall (*N* = 49)
cDOI_MR_ (mm)	
Mean (SD)	7.22 (6.02)
Median [Min, Max]	6.03 [0, 30.0]
Missing	10 (20.4%)
cDOI_IOUS_ (mm)	
Mean (SD)	7.00 (5.08)
Median [Min, Max]	5.00 [0, 20.0]
Missing	9 (18.4%)
pDOI (mm)	
Mean (SD)	7.27 (7.33)
Median [Min, Max]	6.00 [0, 40.0]
Δ (cDOI_MR_- pDOI)	
Mean (SD)	0.146 (3.55)
Median [Min, Max]	1.04 [− 13.0, 3.70]
Missing	10 (20.4%)
Δ (cDOI_IOUS_-pDOI)	
Mean (SD)	0.328 (4.07)
Median [Min, Max]	0.500 [− 13.0, 10.0]
Missing	9 (18.4%)
Δ (cDOI_MR_-cDOI_IOUS_)	
Mean (SD)	− 0.0947 (2.58)
Median [Min, Max]	0 [− 8.96, 3.00]
Missing	19 (38.8%)

**Table 3:** Spearman correlation analysis of DOI measure among different techniques

	pDOI	DOI_IOUS_	DOI_MR_
DOI_MR_	*R* = 0.83 (0.64–0.94) *p* < 0.0001	*R* = 0.87 (0.74–0.94) *p* < 0.0001	1
DOI_IOUS_	*R* = 0.76 (0.59–0.87) *p* < 0.0001	1	
pDOI	1		

In the entire cohort of patients matched measurements by MR, IOUS and histopathology were not significantly different, testing the null hypothesis of cDOI_MR_-pDOI, cDOI_IOUS_-pDOI and cDOI_MR_-cDOI_IOUS_ to be 0, by the Wilcoxon test (*p* = 0.11, *p* = 0.40 and *p* = 0.88, respectively, Fig. 1d, e and Table 2).

MR properly staged 64% of OCSCC lesions, while IOUS reached a perfect correspondence between cT and pT categories in 50% of patients. Weighted Cohen’s kappa test was applied to assess the agreement between MR or IOUS in allocating tumors to the corresponding final pT category, being the k coefficients 0.53 (95% CI 0.32–0.74, *p* < 0.0001) and 0.35 (95% CI 0.14–0.58, *p* = 0.0015), respectively and in-between MR and IOUS 0.71 (95% CI 0.53–0.90, *p* < 0.0001), thus resulting in a good agreement between the two imaging techniques (Table 4 and Table S1).

**Table 4:** Weighted Cohen’s kappa test results between T categories applying different techniques of DOI measurement

	pT category	cT_IOUS_ category	cT_MR_ category
cT_MR_ category	*k* = 0.53 (0.32–0.74) *p* < 0.0001	*k* = 0.71 (0.53–0.90), *p* < 0.0001	1
cT_IOUS_ category	*k* = 0.36 (0.14–0.58) *p* = 0.0015	1	
pT category	1		

**Fig. 4:**
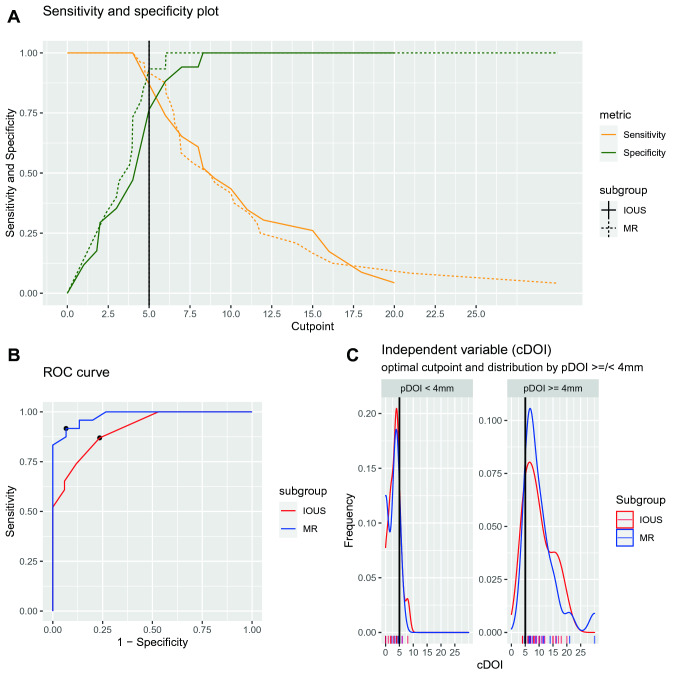
**a** Plot showing the sensitivity and specificity changes, related to the correct detection of a pDOI ≥ 4 mm along with the variation of the cut-off for cDOI_MR_ or cDOI_IOUS._. **b** Receiver operating characteristic (ROC) curve of cDOI_MR_ of cDOI_IOUS_ for pDOI ≥ 4 mm prediction showing the cut-off point of 5 mm for both techniques, by maximization of Youden’s index. **c** Plots showing the distribution of cDOI_MR_ and cDOI_IOUS_ in pDOI ≥ or < 4 mm groups, black vertical line at 5 mm of cDOI

Testing the detection of patients amenable to an elective neck dissection (pDOI ≥ 4 mm), both MR and IOUS obtained good results with a sensitivity of 100% and 100%, respectively; specificity of 73% and 47%, positive predictive value of 86% and 72%, negative predictive value of 100% and 100% and accuracy of 90% and 78%, as reported with full details in Table 5 and Table S2. Furthermore, searching for the best cut-off of cDOI for a predicted pDOI ≥ 4 mm, for both radiological technique it was ≥ 5 mm, by Youden’s index maximization (Fig. 4). The diagnostic test was satisfactory both for MR and IOUS with an Area under the curve (AUC) of 0.92 and 0.82, respectively; a sensitivity of 92% and 87% and a specificity of 93% and 76%, respectively (Fig. 4).

**Table 5:** Diagnostic test results of MR of IOUS assessment of DOI for the prediction of a pDOI ≥ 4 mm

	MR % (CI_95%_)	IOUS % (CI_95%_)
Sensitivity	100 (86–100)	100 (85–100)
Specificity	73 (45–92)	47 (23–72)
Positive predictive value	86 (67–96)	72 (53–86)
Negative predictive value	100 (72–100)	100 (63–100)
Accuracy	90 (76–97)	78 (62–89)

## Discussion

We conducted this prospective study with the specific aim of investigating the performance of the emerging technique of intraoral ultrasonography (IOUS), compared to the gold standard represented by the magnetic resonance (MR) for the DOI evaluation in OCSCC. A similar investigation has been recently conducted by Noorlag and colleagues [13]; though, in their paper radiological TT values (instead of DOI values) from T1-T2 tongue cancers were compared with histopathological DOI.

The data herein show that both cDOI measured by IOUS and MR highly correlate with the pathological result (pDOI), even though MR obtained the best performance with a *R* of 0.83 compared to 0.76 of IOUS as shown in Fig. 3. Similar results were obtained comparing the agreement of a correct cT category allocation, compared to the pT one, with a *k* = 0.53 for MR and *k* = 0.36 for IOUS. These differences could be due to the limits of IOUS to be a new and closely operator-dependent tool. Due to this fact, long-lasting and specific experience in head and neck US is needed to achieve optimal measurement of cDOI in OCSCC. On the contrary IOUS benefits of lower costs and easy application. Investigating the paired differences between the MR or IOUS results and the final pathological measure, matched measurements were not significantly different, meaning the absence of systematic over- or under-estimation of the pDOI for both radiological techniques (Fig. 1).

Nowadays, DOI should be considered as the most trustworthy criterion to be correlated with the threat of regional metastasization and prognostic outcomes in OCSCC. From the therapeutic point of view, cT1 and selected cT2 with cDOI < 10 mm [3], without major infiltration of the extrinsic musculature of the tongue, can be safely removed transorally. By contrast, for more advanced T categories, in the last two decades there has been progressive abandonment of circumferential and/or cuneiform resections in favor of longitudinal compartmental resection to ensure the best loco-regional control and functional outcomes [14–17]. Moreover, cDOI, especially for cT1-cT2N0 lesions, represents an essential prognosticator in deciding whether to perform a simultaneous elective neck dissection or to delay it after the definitive histopathological report [18, 19]. It is well established that the probability of having occult nodal metastases in regional lymph nodes is mainly related to DOI. Mohit-Tabatabai and colleagues [20] and Spiro and colleagues [21] first applied Breslow's hypothesis [22] with reference to the correlation between lymph node involvement and DOI in OCSCC. Nonetheless, to date, controversies still remain about the proper DOI cut-off for a clinically relevant risk of occult nodal metastases. Data from the literature have suggested that a cut-off could be set at 4 mm: consequently, cN0 patients with DOI < 4 mm could be safely spared from elective neck dissection [5–7]. Even if histopathological measurement of DOI remains the standard in the choice for prophylactic neck dissection in early OCSCC, its accurate preoperative measurement allows performing, in selected cases, an elective neck dissection together with tumor resection, thus reducing the number of inappropriate overtreatments or two-step therapeutic approaches (i.e. tumor resection first and delayed neck dissection later). In our series both the evidence of a cDOI_MR_ or cDOI_IOUS_ ≥ 4 mm obtained good results from the diagnostic test for the prediction of a pDOI ≥ 4 mm with an overall accuracy of 90% and 78%, respectively, and a transversal Sensitivity and Negative predictive value of 100% for both techniques, meaning the correct identification of all patients truly amenable to an elective neck dissection and the almost perfect guarantee that a cDOI < 4 mm corresponds to a pDOI < 4 mm too. Morover, investigating the best cut-off of cDOI_MR_ or cDOI_IOUS_ for the correct prediction of a pDOI ≥ 4 mm this was 5 mm for both techniques, as shown in Fig. 4.

Furthermore, the role of pDOI as an independent indication for postoperative RT in early OCSCC still remains controversial [23]. In a recent multicenter study aimed at demonstrating if an increase in DOI parallels significant prognostic deterioration in patients with early OCSCC with no other adverse pathological characteristics, the authors reported that DOI was profoundly connected with other risk factors including involved or close surgical margins, dimensions of the primary tumor, pathological nodal staging, and extra-nodal extension [24]. Moreover, in a recent analysis of 1200 patients affected by OCSCC, the authors underlined the importance of DOI > 10 mm as an adjunctive indicator to deliver postoperative RT, even if it is not explicitly taken into account by NCCN guidelines [25].

All these findings clearly suggest the importance of accurate preoperative prediction of DOI in early-intermediate OCSCC. So far, MR has been shown to be the most accurate instrument for loco-regional staging of OCSCC, with a sensitivity of 94% according to the literature [26]. To date, a clear consensus in the literature concerning the best MR sequence with which to measure DOI is still lacking. Preda et al. [27] reported that contrast administration enhances tumor differently, depending on its size and vascularization, while in T2-weighted images lesions are predictably hyperintense. Some authors reported the use of different sequences to obtain the best measurement of the tumor: axial gadolinium-enhanced T1 sequence specifically for DOI, axial and coronal T2 and post-contrast T1 for the entire size of the lesion are proposed by Goel et al. [28]. On the other hand, Murakami et al. reported in their work that the selection of the optimal measurement protocol should be made on a case-by-case basis [29]. A significant issue that may negatively influence the accuracy of MR measurements is related to image quality due to the presence of possible motional artifacts. To resolve this issue, some authors [30] proposed the use of the BLADE sequence to reduce artifacts due to motion, pulsation [31], and dental implants [32].

Better performance of 3.0-T over 1.5-T MR is still a matter of debate in the literature. According to Moreno et al. [33] and Lu et al. [34], an expected signal-to-noise ratio increase proportional to the magnetic field strength is the most interesting characteristic of 3.0-T MR, but other features such as increased T1 relaxation time, decreased T2 relaxation time, increased magnetic susceptibility contrast, and increased spectral resolution for MR spectroscopy may also furnish important benefits. In contrast, Neumann et al. [35] noted some critical issues of 3.0-T MR due to the higher magnetic susceptibility that, according to the authors, led to possible spatial distortion. Singh et al. [36] reported the good agreement (*K* = 0.79) for T staging between MR and histopathology, with a change in the final pT category for only 14% of patients.

Baek et al. [37] reported on the utility of IOUS in prognosticating pathologic OCSCC TT. Furthermore, according to the authors, even if both CT and MR have some limitations in the evaluation of tongue cancers, MR gives more detailed information about soft tissue involvement compared to CT. According to Yesuratman et al. [18], preoperative IOUS shows a high correlation index with histopathology (*r* = 0.80), while MR demonstrates only a moderate one (*r* = 0.69). One of the major confounding factors in diminishing the trustworthiness of preoperative radiological evaluation is a recent biopsy. It is arduous for IOUS and MR to distinguish post-biopsy edema from squamous dysplasia and/or invasive SCC. In general, inflammation surrounding the tumor could blur the boundaries that are observed on MRI T2-weighted sequences, leading to a possible overestimation of DOI [29]. On the contrary, IOUS can provide some advantages due to the possibility of changing the orientation of the probe along different planes, even through the skin of the face if needed. Moreover, as practical tools to assess thin muscles and buccal fat layers, it can be helpful to ask the patient to protrude and move the tongue or swollen cheek. Nonetheless, to date, some disagreements persist with regards to the best IOUS probe, the ideal probe frequency, and proper usage. In this regard, we chose a T-shape linear probe for the present study, following the suggestions of Iida and coworkers [38]. According to those authors, the best method to preoperatively measure DOI is the aforementioned probe inserted into a rubber sheath and filled with water. In this way, it is possibly best to outline the real 3D extent of the tumor, mainly because the same evaluation may also be reproduced in the operating theater, just before the intervention.

The impossibility of attaining all oral subsites and the obstacle represented by adjacent bony structures appear to be the most considerable limitations of IOUS evaluation. As a consequence, tumors located in the posterior third of the mobile tongue are not easily accessible to perpendicular evaluation. Other IOUS technical issues are related to the fact that the probe should be kept in tight contact with the mucosal surface to be assessed to maximize the interface between them, trying not to exert an excessive pressure that could possibly distort or modify the aspect of the tumor with a consequent risk of underestimation of DOI. Moreover, IOUS is a live examination that is strictly dependent on the operator: as a consequence, variable levels of experience may give different information. As a somewhat new diagnostic tool, IOUS needs a proper learning curve to understand the adequate pressure to apply to tissues, the way to move the probe, and how to reach the more posterior subsites of the oral cavity. Finally, the same exam cannot be subsequently reassessed by a second operator nor visually aid surgeons before or during surgery.

Herein, both MR and IOUS had good correlation with histopathological findings and between them. For these reasons, we firmly believe that IOUS might be safely used, even alone, for preoperative staging of early OCSCC situated in the anterior half of the oral cavity.

The main strength of our study is represented by the homogeneous evaluation of the entire cohort of patients by the same two experienced head and neck radiologists. Moreover, the majority of patients included were evaluated by both preoperative MR and IOUS, allowing direct and fruitful comparison of the two radiological tools on the same lesion. The main concern of our findings is that our cohort of patients is relatively restricted and that the mean time dividing radiological and histopathological examination was almost 20 days.

## Conclusion

At present, MR is considered the first-choice radiological examination for preoperative diagnostic assessment of head and neck SCCs, except for the larynx and hypopharynx. With MR, the radiologist is able to analyze neoplastic extension, intratumoral vascularization, tumor borders, and intracranial and/or perineural spreads. Its main limitations are that it is expensive, requires substantial time to perform a proper exam, and is impossible to perform in non-cooperative or claustrophobic patients, or in those with metallic prostheses and pacemakers. By contrast, the advantages of IOUS are related to the fact that is a fast and high-resolution examination, less invasive, more cost-effective, and requires less compliance by the patient. On the other hand, it remains highly operator-dependent and cannot be considered as the best radiological examination for lesions in close proximity to bony structures or located in the posterior half of the oral cavity. Notwithstanding, from our data it is possible to infer a good performance of IOUS and a good agreement with the current gold standard examination (MR). From these results, physicians may be encouraged to use IOUS as a complementary evaluation, being a less expensive and faster tool in the preoperative diagnostic work-up of early-intermediate anterior OCSCC.

## Electronic supplementary material

Below is the link to the electronic supplementary material.

Supplementary file1 (DOCX 14 kb)
