# Supplementary material for: Intraoral ultrasonography in the assessment of DOI in oral cavity squamous cell carcinoma: a comparison with magnetic resonance and histopathology
Source: Eur Arch Otorhinolaryngol. 2020 Oct 21;278(8):2943–52. doi: 10.1007/s00405-020-06421-w (PMC8266699; doi:10.1007/s00405-020-06421-w)
Supplement: Supplementary file 1 — Supplementary file1 (DOCX 14 kb) [file 405_2020_6421_MOESM1_ESM.docx]

**Supplementary Tables**

**Table S1:** Contingency tables showing the pT category distribution compared to the cT_MR_ and cT_IOUS_ ones. *Legend*: the blue font indicates the over-estimated patients; the orange one indicates the under-estimated patients.

|  | **pT1 (N=15)** | **pT2 (N=21)** | **pT3 (N=13)** | **Overall (N=49)** |
| --- | --- | --- | --- | --- |
| **cT_MR_ category** |  |  |  |  |
| cT1 | **9 (60.0%)** | **5 (23.8%)** | **1 (7.7%)** | 15 (30.6%) |
| cT2 | **2 (13.3%)** | **10 (47.6%)** | **3 (23.1%)** | 15 (30.6%) |
| cT3 | **0 (0%)** | **3 (14.3%)** | **6 (46.2%)** | 9 (18.4%) |
| Missing | 4 (26.7%) | 3 (14.3%) | 3 (23.1%) | 10 (20.4%) |
| **cT_IOUS_ category** |  |  |  |  |
| cT1 | **8 (53.3%)** | **8 (38.1%)** | **1 (7.7%)** | 17 (34.7%) |
| cT2 | **4 (26.7%)** | **7 (33.3%)** | **4 (30.8%)** | 15 (30.6%) |
| cT3 | **0 (0%)** | **3 (14.3%)** | **5 (38.5%)** | 8 (16.3%) |
| Missing | 3 (20.0%) | 3 (14.3%) | 3 (23.1%) | 9 (18.4%) |

**Table S2:** Contingency tables showing the frequency of histopathologic pDOI ≥ 4 mm compared to a clinical DOI_MR_ ≥ 4 mm or DOI_IOUS_ ≥ 4 mm pT category distribution compared to the cT_MR_ and cT_IOUS_.

|  | **pDOI ≥ 4 mm** | | **Overall (N=49)** |
| --- | --- | --- | --- |
|  | **no (N=19)** | **yes (N=30)** |  |
| **cDOI_MR_ ≥ 4 mm** |  |  |  |
| no | 11 (57.9%) | 0 (0%) | 11 (22.4%) |
| yes | 4 (21.1%) | 24 (80.0%) | 28 (57.1%) |
| Missing | 4 (21.1%) | 6 (20.0%) | 10 (20.4%) |
| **cDOI_IOUS_ ≥ 4 mm** |  |  |  |
| no | 8 (42.1%) | 0 (0%) | 8 (16.3%) |
| yes | 9 (47.4%) | 23 (76.7%) | 32 (65.3%) |
| Missing | 2 (10.5%) | 7 (23.3%) | 9 (18.4%) |
